# Atypical idiopathic intracranial hypertension presenting as cyclic vomiting syndrome: a case report

**DOI:** 10.1186/s13256-021-03068-x

**Published:** 2021-08-31

**Authors:** Nafee T. Talukder, Amanda H. Clorfeine, Moira K. Black, Shade B. Moody

**Affiliations:** 1 Department of Neurology, Children’s Memorial Hermann Hospital, 6410 Fannin St., Ste 1014, Houston, TX 77030 USA; 2 University of Texas Health Science Center, Houston, TX USA

**Keywords:** IIH, Cyclic vomiting syndrome, Pediatrics, Rare associations, Episodic vomiting

## Abstract

**Background:**

Idiopathic intracranial hypertension is a disorder of increased intracranial pressure in the absence of cerebrospinal outflow obstruction, mass lesion, or other underlying cause. It is a rare phenomenon in prepubertal children and is most typically found in women of childbearing age. The classic presentation consists of headaches, nausea, vomiting, and visual changes; however, children present more atypically. We report a case of idiopathic intracranial hypertension in an otherwise healthy, 4-year-old child with atypical symptoms resembling those of cyclic vomiting syndrome.

**Case presentation:**

A 4-year-old Caucasian, otherwise healthy, male child presented to our emergency department with episodic intermittent early-morning vomiting occurring once every 1–3 weeks without interepisodic symptoms, starting 10 months prior. With outpatient metabolic, autoimmune, endocrine, allergy, and gastroenterology work-up all unremarkable, he was initially diagnosed with cyclic vomiting syndrome. Discovery of mild optic nerve sheath distension on magnetic resonance imaging of the brain 10 months after symptom onset led to inpatient admission and a lumbar puncture notable for an opening pressure of 47 mmHg, with normal cell count and protein levels. He had no changes in visual acuity or optic disc edema on dilated fundoscopic examination. The patient was started on acetazolamide, with resolution of episodic emesis at his last follow-up visit 12 weeks after discharge.

**Conclusions:**

Idiopathic intracranial hypertension presents atypically in prepubescent children, with about one-fourth presenting asymptomatically, and only 13–52% presenting with “classic” symptoms. With a prevalence of only 0.6–0.7 per 100,000, much remains unknown regarding the underlying pathophysiology in this demographic. Cyclic vomiting syndrome, however, has a much higher prevalence in this age group, with a prevalence of 0.4–1.9 per 100. It is thought to be an idiopathic, periodic disorder of childhood, often linked to neurological conditions such as abdominal migraines, epilepsy, mitochondrial disorders, and structural lesions such as chiari malformation and posterior fossa tumors. While cyclic vomiting syndrome is thought to have a benign course, untreated idiopathic intracranial hypertension can have long-term detrimental effects, such as visual loss or even blindness. We present a case of idiopathic intracranial hypertension presenting with symptoms resembling cyclic vomiting syndrome in a 4-year-old child, diagnosed 10 months after initial onset of symptoms. We aim to demonstrate the need for a high level of clinical suspicion and the need for further investigation into underlying pathophysiology in this vulnerable population.

## Background

Idiopathic intracranial hypertension (IIH) is a disorder of increased intracranial pressure in the absence of cerebrospinal fluid (CSF) outflow obstruction, masses, or other secondary etiology [1]. Classically, it is thought to occur in women of childbearing age with presenting symptoms such as headache, nausea, vomiting, and visual changes. The prevalence of IIH in the prepubescent population is lower. This fact, in combination with their atypical presentations, results in lower low levels of clinical suspicion, causing the diagnosis to often be missed or delayed. One-fourth of this population may be asymptomatic at time of diagnosis, with only 13–52% presenting with classic symptoms [2–4]. Even when suspected, pediatric patients are often found to have lower opening pressures (OP) during lumbar puncture (LP), lower grades of optic nerve edema, and less discernible findings on magnetic resonance imaging (MRI) [5]. Early diagnosis and intervention is crucial; if left untreated, severe optic disc edema may, ultimately, lead to permanent vision loss [6, 7].

We present the case of a prepubescent boy with recurrent, stereotypic, early-morning vomiting episodes without associated headaches, nausea, visual deficits, or papilledema. While vomiting is a common symptom of increased intracranial pressure (ICP), to our understanding, no cases have been reported in the literature of IIH presenting with episodic vomiting resembling that of cyclic vomiting syndrome (CVS).

## Case presentation

**Fig. 1:**
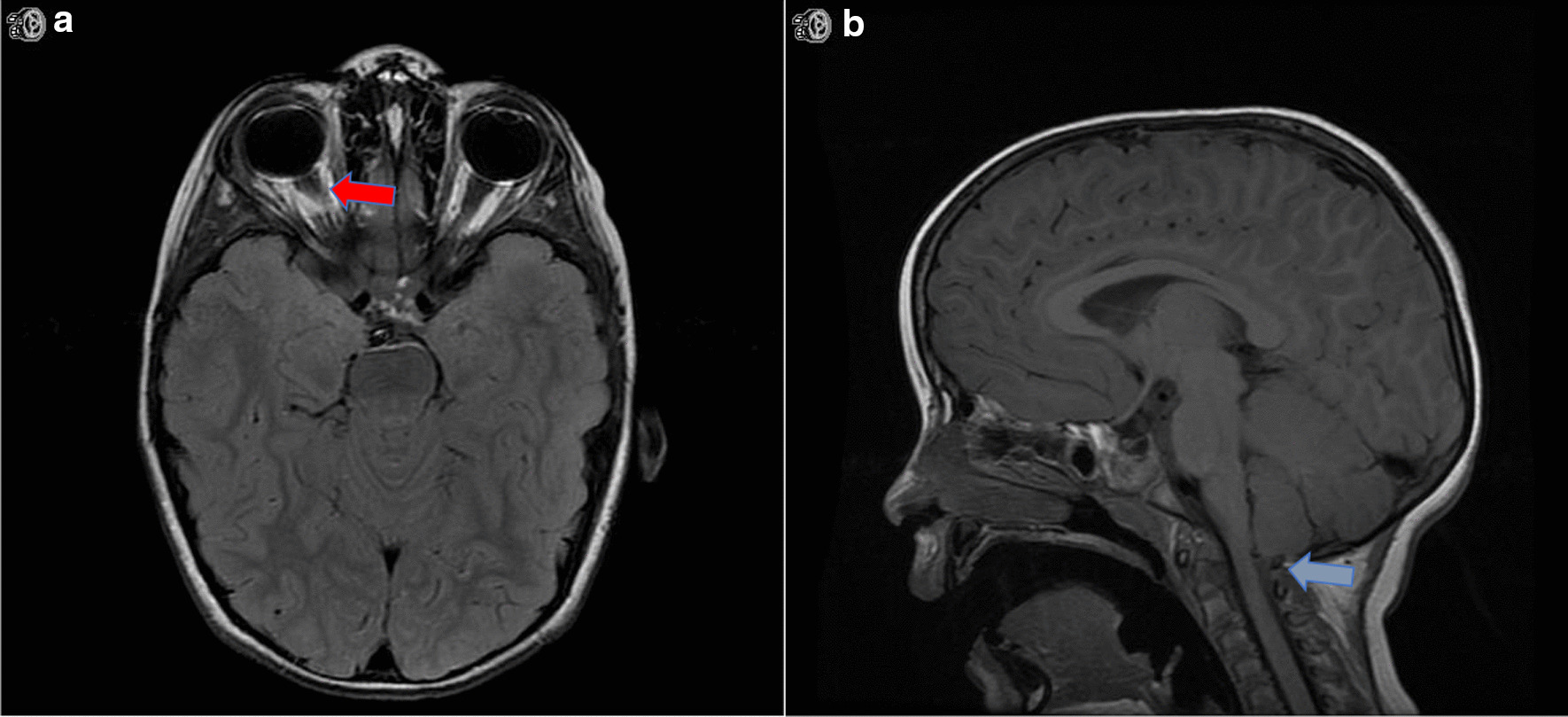
Neuroimaging demonstrating increased intracranial pressure. **A** Magnetic resonance imaging axial T2 FLAIR sequence displaying optic nerve sheath distension without flattening of the globes (red arrow). **B** Magnetic resonance imaging sagittal T1 sequence displaying tip of the cerebellar tonsils at the level of foramen magnum causing mild effacement of the subarachnoid fluid spaces across the foramen magnum (blue arrow)

A 4-year-old Caucasian male born at term without complications, otherwise healthy, was sent to our emergency department (ED) by his gastroenterologist (GE) owing to MRI findings of mild optic nerve sheath distension. He was being seen by GE for intermittent episodes of early-morning vomiting starting 10 months prior, occurring once every 1–3 weeks only upon awakening in the morning and with return to his usual state of health within 1–2 hours following these events. His parents denied other associated symptoms, such as abdominal pain, visual impairment, loss of balance, or changes in bowel and bladder continence. They endorsed that he may have had a few headaches in the past 10 months, lasting brief seconds while playing, without temporal correlation to his events. Upon onset, he was initially worked up by his pediatrician, with hematologic, metabolic, hormonal, allergic, and immunologic work-up all unremarkable. Pediatric GE performed an esophagogastroduodenoscopy (EGD) without any notable masses or lesions, and multisite biopsy without inflammatory changes. Given his strong family history for CVS (five paternal cousins) and continued stereotypic episodes without change in frequency or character, he was ultimately diagnosed with CVS and initiated on antiemetic therapy. Unlike his cousins, however, his symptoms did not subside. His pediatrician obtained an MRI brain with contrast, which was only notable for mild bilateral optic nerve sheath distension without masses, globe flattening, or other signs suggestive of increased intracranial pressure (Fig. 1), prompting his urgent referral to our ED.

At presentation, the patient was in his usual state of health: active, curious, socially engaging, and running back and forth through the examination room. His last episode of vomiting occurred about 2 weeks prior, and he had been at his baseline since. Initial metabolic, hematologic, and infectious work-up were all unremarkable. Severe acute respiratory syndrome coronavirus 2 (SARS-CoV-2) was negative. Neurological examination was without focal deficits, with his mental status at baseline, speaking in full coherent sentences, and asking inquisitive questions. Cranial nerves were without deficit and without evidence of nystagmus or visual field cuts. Visual acuity was equal bilaterally without impairment. Dilated fundus examination by ophthalmology demonstrated healthy pink optic nerves bilaterally without disc edema. LP demonstrated an elevated OP of 47 mmHg with normal glucose, protein, and cell count. Magnetic resonance venography (MRV) of the brain yielded no venous sinus thrombosis or stenosis. He was initiated on carbonic anhydrase inhibitor (CA-I) therapy with acetazolamide, and discharged home with close outpatient follow-up. At outpatient follow-up visits, most recently 12 weeks following discharge, his mother endorsed significant improvement in his mood and resolution of his early-morning episodic vomiting, stating this was the best she had seen him in a year.

## Discussion

CVS is an idiopathic chronic periodic disorder of childhood, with a prevalence of about 0.4–1.9 per 100 [8]. The diagnosis is clinical and is commonly based on ICHD-III, The North American Society for Pediatric Gastroenterology, Hepatology, and Nutrition consensus statement [9], and/or the ROME IV criteria [10]. The hallmark consists of stereotypical episodes of vomiting, occurring at least 1 week apart, and in the absence of positive findings on laboratory, radiographic, or endoscopic testing [10]. While many mimickers of CVS are noted in the literature, such as abdominal migraine, demyelinating disorders, intracranial masses, GI disorders, and mitochondrial disorders [8], no reported cases were found of IIH mimicking CVS. In comparison, the prevalence of IIH in the prepubescent population is far scarcer, with an overall prevalence of about 0.6–0.7 per 100,000 [2]. The underlying pathophysiology is thought to differ in this population, compared with the classic demographic of women of childbearing age, as it is not typically associated with obesity and can present atypically, with the classic daily diffuse, non-pulsating headache exacerbated with Valsalva only found in about 37%, nausea and vomiting in only 13–52%, and transient visual deficits in only 42% [3, 4]. Cranial neuropathies have often been reported, most commonly cranial nerve (CN) VI (abducens nerve) palsies [2], but also hemifacial spasm, CN IV (trochlear nerve) palsies, generalized ophthalmoparesis [11], and a single reported case of isolated CN VII (facial nerve) palsy [12].

While IIH is traditionally believed to affect obese women of childbearing age, an increase in reported cases in the pediatric population calls for a shift in how this disorder is approached [13]. The commonly accepted pathophysiology involves an imbalance in CSF production relative to its resorption [14]; however, in children it is thought to be more complex and may differ based on stage of development. Pediatric IIH can be broken down into three distinct subgroups: young prepubertal patients with normal height and body mass index (BMI), early adolescents who are overweight and taller, and late-pubertal adolescents who are of normal height but are obese [15]. In early and late adolescence, adiposity and its effects on hormonal regulation and glucocorticoid metabolism [16] is thought to underpin its etiology. However, in prepubertal children, IIH tends to have an equal distribution between males and females, and adiposity does not appear to be a risk factor [17, 18]. A possible explanation may be increased production of CSF in this age group as demonstrated by decreased CSF protein concentration [14]. Increased intracranial pressure in this age group, however, is predominantly due to secondary causes, such as cerebral venous sinus thrombosis or stenosis, hypervitaminosis A, trisomy 21, endocrine disorders, anemia, tetracycline use, trans-retinoic acid use, and chronic steroid withdrawal [11]. Within this prepubertal subgroup, neuroimaging findings of increased ICP differs among cases of IIH and those with secondary etiologies, suggesting a more insidious pathology [19].

Quincke’s 1893 “meningitis serosa” publication formally defined IIH in its description of increased intracranial pressure without a brain tumor [11]. A 1937 report on a series of 22 patients, by Dandy *et al*., established the framework for its initial diagnostic criteria [20]. These criteria have undergone multiple modifications in time with advancements in medical diagnostic capabilities. The most recent version of the modified Dandy criteria consists of: (1) signs and symptoms of increased ICP (papilledema, transient visual obscurations, nausea/vomiting, and headaches); (2) lack of focal neurologic signs aside from CN VI palsies; (3) CSF OP of greater than or equal to 25 mmHg, without chemical or cytologic abnormalities; and (4) neuroimaging excluding cerebral venous sinus thrombosis or masses [21]. These parameters have classically been based on analysis of post-pubertal populations. A lack of consensus exists on criteria that fully encompass the prepubescent population [13]. Friedman *et al*. suggested a revised diagnostic criterion that excludes the incorporation of clinical symptoms and relies on objective diagnostic and radiographic findings [22]. This approach would further risk overlooking the large proportion of the prepubertal population that present with atypical objective findings, such as a lower OP on LP, lower incidence of globe flattening, and lower grades of optic nerve sheath distension and edema [2, 5, 23]. Based on presenting symptoms and clinical findings, the above-mentioned diagnostic criteria in isolation would miss prepubescent patients presenting similarly to ours.

CA-I is the mainstay of medical treatment for IIH, as it targets CSF production [21]. Among this class, acetazolamide is most frequently utilized. Topiramate, a classic antiepileptic medication with CA-I properties, is commonly used in patients with concurrent headaches [2]. Mixed findings are reported in the literature as to whether acetazolamide or topiramate is the more efficacious drug of choice [6, 7]. When attributed to obesity, weight loss becomes crucial in management, with 6% loss of total body weight found to reduce optic disc edema [6]. In cases of significant vision loss with papilledema and headache, CSF diversion procedures with ventriculoperitoneal shunts (VPS), lumboperitoneal shunts (LPS), and optic nerve sheath fenestration (ONSF) are utilized [2]. To date, no large, randomized control trials or meta-analyses have been completed to formally establish a treatment protocol [21].

## Conclusions

We present a unique manifestation of IIH in a 4-year-old boy with stereotypic early-morning vomiting resembling CVS without classic associated symptoms of headache, visual changes, or cranial neuropathies. His prompt response to minimal doses CA-I therapy was consistent with prepubescent IIH cases in the literature, where shorter duration and smaller dosages of therapy are required for symptom management [5, 7]. This response favors proposed theories of CSF overproduction in prepubescent IIH, but fails to explain the transient, episodic, yet stereotypical nature of our patient’s presenting symptoms. We present this case with the aim to call for higher level of clinical suspicion for atypical presentations of IIH in this vulnerable population in preventing lifelong harm. We emphasize the need for a deeper understanding of its underlying pathophysiology in development of more precise diagnostic criteria and treatment protocols.

## Data Availability

Not applicable to this manuscript type.

## Abbreviations

IIH: Idiopathic intracranial hypertension
CVS: Cyclic vomiting syndrome
LP: Lumbar puncture
MRI: Magnetic resonance imaging
MRV: Magnetic resonance venography
EGD: Esophagogastroduodenoscopy
FLAIR: Fluid-attenuated inversion recovery
ED: Emergency Department
CSF: Cerebrospinal fluid
CN: Cranial nerve
VPS: Ventriculoperitoneal shunt
LPS: Lumboperitoneal shunt
ONSF: Optic nerve sheath fenestration
